# CMR-derived left ventricular intraventricular pressure gradients identify different patterns associated with prognosis in dilated cardiomyopathy

**DOI:** 10.1093/ehjci/jead083

**Published:** 2023-05-03

**Authors:** Jacqueline L Vos, Anne G Raafs, Michiel T H M Henkens, Gianni Pedrizzetti, Caroline J van Deursen, Laura Rodwell, Stephane R B Heymans, Robin Nijveldt

**Affiliations:** Department of Cardiology, Radboud University Medical Center, Geert Grooteplein 10, 6525 GA, Nijmegen, The Netherlands; Research Institute Maastricht (CARIM), Maastricht University Medical Centre, P. Debyelaan 25, 6229 HX Maastricht, The Netherlands; Research Institute Maastricht (CARIM), Maastricht University Medical Centre, P. Debyelaan 25, 6229 HX Maastricht, The Netherlands; Department of Engineering and Architecture, University of Trieste, Via Alfonso Valerio, 6/1, 34127 Trieste, Italy; Department of Biomedical Engineering, University of California, 402 E Peltason Dr, Irvine, CA 92617, USA; Department of Cardiology, Radboud University Medical Center, Geert Grooteplein 10, 6525 GA, Nijmegen, The Netherlands; Health Evidence, Section Biostatistics, Radboud Institute for Health Sciences, Geert Grooteplein 10, 6525 GA, Nijmegen, The Netherlands; Research Institute Maastricht (CARIM), Maastricht University Medical Centre, P. Debyelaan 25, 6229 HX Maastricht, The Netherlands; Department of Cardiovascular Research, University of Leuven, Herestraat 49, 3000 Leuven, Belgium; Department of Cardiology, Radboud University Medical Center, Geert Grooteplein 10, 6525 GA, Nijmegen, The Netherlands

**Keywords:** CMR, feature tracking, intraventricular pressure gradient, left atrial strain, left ventricular strain, dilated cardiomyopathy

## Abstract

**Aims:**

Left ventricular (LV) blood flow is determined by intraventricular pressure gradients (IVPG). Changes in blood flow initiate remodelling and precede functional decline. Novel cardiac magnetic resonance (CMR) post-processing LV-IVPG analysis might provide a sensitive marker of LV function in dilated cardiomyopathy (DCM). Therefore, the aim of our study was to evaluate LV-IVPG patterns and their prognostic value in DCM.

**Methods and results:**

LV-IVPGs between apex and base were measured on standard CMR cine images in DCM patients (*n* = 447) from the Maastricht Cardiomyopathy registry. Major adverse cardiovascular events, including heart failure hospitalisations, life-threatening arrhythmias, and sudden/cardiac death, occurred in 66 DCM patients (15%). A temporary LV-IVPG reversal during systolic–diastolic transition, leading to a prolonged transition period or slower filling, was present in 168 patients (38%). In 14%, this led to a reversal of blood flow, which predicted outcome corrected for univariable predictors [hazard ratio (HR) = 2.57, 95% confidence interval (1.01–6.51), *P* = 0.047]. In patients without pressure reversal (*n* = 279), impaired overall LV-IVPG [HR = 0.91 (0.83–0.99), *P* = 0.033], systolic ejection force [HR = 0.91 (0.86–0.96), *P* < 0.001], and E-wave decelerative force [HR = 0.83 (0.73–0.94), *P* = 0.003] predicted outcome, independent of known predictors (age, sex, New York Heart Association class ≥ 3, LV ejection fraction, late gadolinium enhancement, LV-longitudinal strain, left atrium (LA) volume-index, and LA-conduit strain).

**Conclusion:**

Pressure reversal during systolic–diastolic transition was observed in one-third of DCM patients, and reversal of blood flow direction predicted worse outcome. In the absence of pressure reversal, lower systolic ejection force, E-wave decelerative force (end of passive LV filling), and overall LV-IVPG are powerful predictors of outcome, independent of clinical and imaging parameters.

## Graphical Abstract

**Graphical Abstract:**
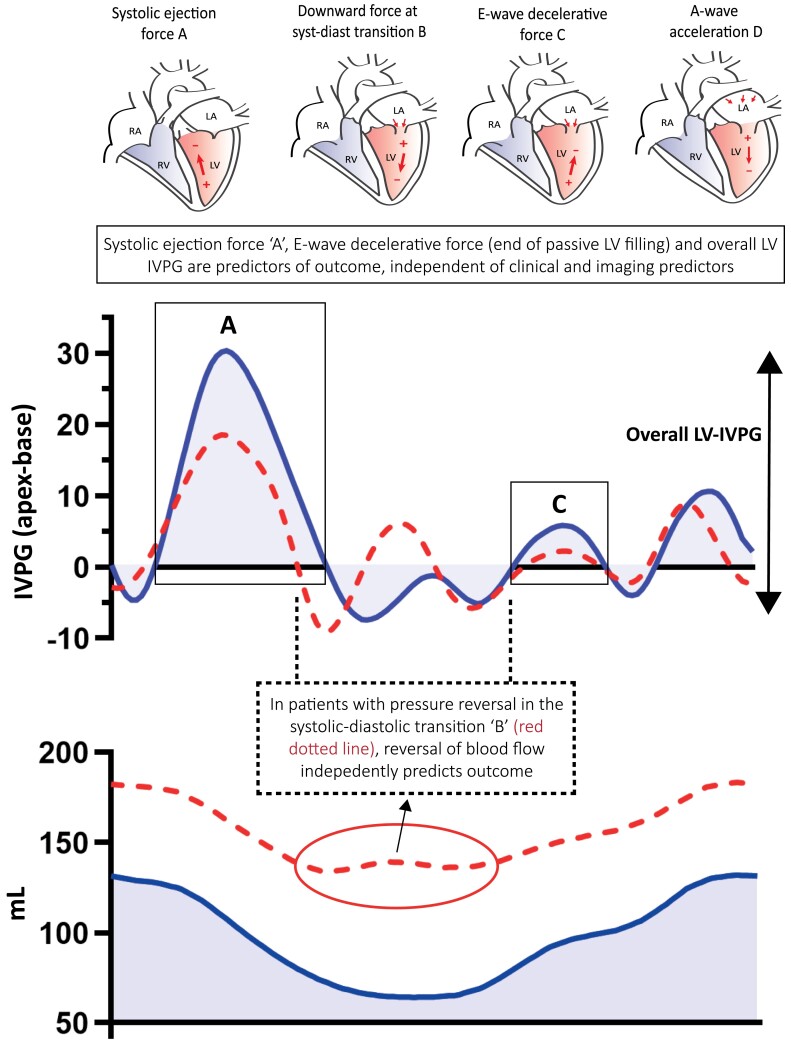

## Introduction

In dilated cardiomyopathy (DCM), characterized by reduced systolic cardiac function and left ventricular (LV) dilation, cardiovascular magnetic resonance (CMR) imaging is recommended and can be used for both diagnostic and prognostic purposes.^1,2^ Myocardial functional parameters, such as left ventricular ejection fraction (LVEF) or global longitudinal strain (GLS), and late gadolinium enhancement (LGE), are strong prognostic markers.^3^ Nonetheless, it remains hard to distinguish which patients will remain stable for years and who will suffer from a detrimental course of the disease.

Cardiac fluid dynamics are a topic of interest in research^4–7^ and may be of potential value in DCM patients. Intraventricular pressure gradients (IVPG) between apex and base in the LV drive blood flow. Alterations in IVPG, and thus blood flow, initiate ventricular adaptation, and hence, changes in IVPG precede changes in cardiac function and geometry.^7^ IVPG are generated by myocardial properties, such as ventricular contraction (ejection phase), ventricular relaxation and recoil (early filling), atrial contraction (late filling), and pressure in the large vessels. These IVPGs between apex and base in diastole and in systole are attenuated, or even lost, in heart failure (HF) and myocardial infarction animal studies.^4–6^ However, the invasive and time-consuming aspect of this measurement has restricted it from implementation in clinical practice. Analysis of haemodynamic forces is a novel post-processing analysis that enables estimating LV-IVPG non-invasively on standard CMR cine images.^8^ LV-IVPG were decreased in patients with HF with both reduced and preserved LVEF.^9^ The ability to evaluate diastolic alterations in LV-IVPG would be of special value, since assessment of diastolic function has been a major shortcoming of CMR imaging. Additionally, long-term outcome data of CMR-derived LV-IVPG have not been investigated so far. Therefore, the aim of this study was to evaluate alterations in CMR-derived LV-IVPG and their prognostic value in DCM patients.

## Methods

The Maastricht Cardiomyopathy Registry enrols patients meeting the definition of DCM [i.e. reduced LVEF and increased BSA-indexed LV end-diastolic volume (LVEDVi)].^1^ In accordance with the European Society of Cardiology (ESC) proposal,^10^ exclusion criteria include (i) significant coronary artery disease; (ii) primary valvular disease; (iii) hypertensive or congenital heart disease; (iv) acute myocarditis; (v) arrhythmogenic right ventricular dysplasia; (vi) hypertrophic, restrictive, or peripartum cardiomyopathy; and (vii) storage diseases. Patients with available long-axis cines (2-, 3-, and 4-chamber views) between 2004 and 2019 were selected (see Supplementary data online, *Figure S1*: flow-chart). Patients with atrial fibrillation during CMR were excluded (*n* = 63). All patients underwent an extensive standardized diagnostic work-up, including blood sampling, electrocardiography, and physical examination. The presence of conduction delay was defined as QRS >120 ms and was either marked as typical left bundle branch block (LBBB) or non-typical LBBB based on the ESC criteria for advanced LBBB.^11^ The study was performed according to the Helsinki Declaration, and the local ethical review board approved the study. All patients gave written informed consent.

### Follow-up

Follow-up data were collected using medical records, municipal population register, and/or contact by telephone with general practitioners. Data on sudden or cardiac death, HF hospitalization and life-threatening arrhythmias (LTAs), defined as non-fatal ventricular fibrillation, and/or haemodynamic unstable ventricular tachycardia with or without cardioverter-defibrillator shock were collected. The primary endpoint was a combination of sudden or cardiac death, HF hospitalization, and LTAs.

### CMR acquisition and analysis

CMR imaging was performed on a 1.5T system (Intera, Philips Medical Systems, Best, The Netherlands), including cine and LGE imaging in identical slice positions in short-axis (covering the entire LV) and long-axis orientations (including 2-, 3-, and 4-chambers). All post-processing analyses were performed using Medis Suite MR software (Medis Medical Imaging BV, Leiden, The Netherlands). LV and right ventricular (RV) volumes and LV mass were measured, and LV/RV ejection fraction were calculated. LGE images were obtained 10–15 min after administration of a gadolinium-based contrast agent, using a 2D segmented inversion-recovery gradient-echo pulse sequence. LV-GLS was measured on long-axis 2-, 3-, and 4-chamber cine images and left atrium (LA) reservoir, conduit and booster strains on long-axis 2- and 4-chamber cine images. Detailed CMR acquisition and analysis protocols are described in the Supplementary data online, *Methods*.

#### CMR LV-IVPG analysis

Haemodynamic force analysis is a method to calculate the global LV-IVPG vector between apex and base. Using the well-known Navier–Stokes equation and aid of the Gauss theorem, the blood velocity, v, can be computed using the force vector integral over the LV boundary [*S (t)*] instead of the internal volume. Thus, it just needs the velocity over the endocardial boundary (derived from the myocardial movement using feature tracking strain), and the blood velocity across the valves (which is calculated from the volumetric changes of the LV and the valve area, using the conservation of mass principle). The mathematical formula is: $F(t)=\rho \int \overset{}{\underset{S(t)}{\int }}\;\left[x\left(\frac{\partial v}{\partial t}\cdot n\right)+v\;(v\cdot n)\right]dS$, in which ρ is the blood density (1.0 kg/L), x the three-dimensional co-ordinate of the points surrounding the LV volume, and n the normal unit vector. More details can be found in the Supplementary data online, *Methods* and in recent articles of Vallelonga *et al.*^12^ and Pedrizetti *et al.*^8^

**Figure 1:**
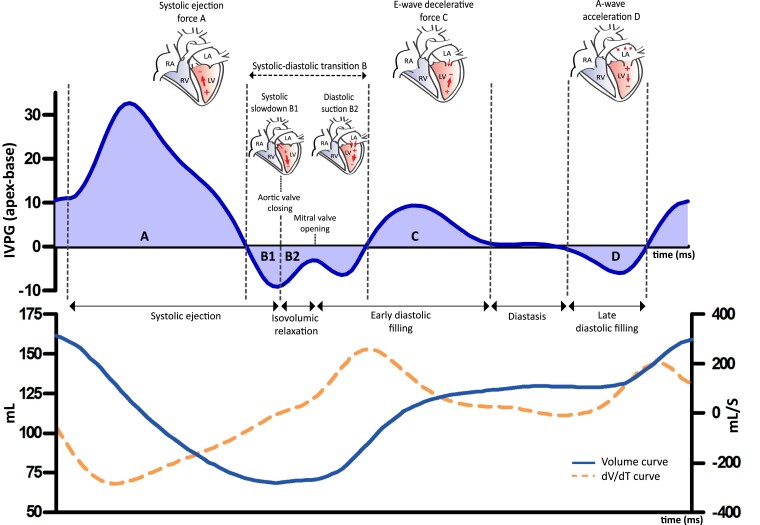
Example of the LV-IVPG curve in a DCM patient, with corresponding volume and dV/dT curve. Depiction of the apex–base LV-IVPG (*y*-axis) over time (*x*-axis, ms) in a DCM patient, with the corresponding volume and *dV*/*dT* curve. If the IVPG is directed from apex to base (apical pressures are higher), it is depicted as a positive wave. If the IVPG is directed from base to apex (basal pressures are higher), it is depicted as a negative wave. These IVPGs drive cardiac blood flow to accelerate or decelerate inward (LV filling) or outward (LV ejection, depicted in the *dV*/*dT* curve) flow. LV contraction drives an IVPG from apex–base (positive wave), exceeding aortic pressure, which drives cardiac blood flow in the aorta. The B-wave consists of two components. At the end of LV contraction, the LV starts to relax and unwind. Aortic pressure starts to exceed LV pressure, decelerating blood flow until the aortic valve closes. This base–apex directed IVPG (negative wave) is the systolic slowdown (B1). B2 starts in the isovolumic relaxation phase. Further unwinding lowers LV pressures until LV pressure is lower than LA pressure, opening the mitral valve and creating a base–apex directed IVPG (negative wave), causing an acceleration of blood flow in the early filling phase, called diastolic suction (B2). Upward mitral annular movement causes additional filling, until the point that LV pressures exceed LA pressure which decelerates blood flow, creating an apex–base IVPG (positive wave): the E-wave deceleration (C). Passive filling ends when pressures equilibrate between apex and base, known as the diastasis. In late diastole, atrial contraction drives a second base–apex IVPG (negative wave), the A-wave acceleration (D).

The IVPG-time curve represents the IVPG directed from apex to base throughout the cardiac cycle. The overall LV-IVPG (throughout the whole cardiac cycle), A-, B-, C-, and D-waves evaluated in this study are illustrated in *Figure 1* and in the tutorial video of the IVPG analysis in the Supplementary Material (see Supplementary data online, *Video S1*, modified from Vos *et al.*^13^). All CMR analyses were performed by two trained independent investigators (J.V. and A.R.), blinded to outcome and supervised by a level III CMR physician with >15 years of experience (R.N.). To evaluate the interobserver variability of the LV-IVPG analysis, the analyses were repeated in 20 CMR scans by two investigators. The interobserver variability was good to excellent for all LV-IVPG measurements (see Supplementary data online, *Table S5*).

### Statistical analysis

Categorical variables are presented as number (percentages). Normality of continuous variables was assessed visually using Q–Q plots and histograms and displayed as mean ± standard deviation when normally distributed, or median [interquartile range] if not. Categorical variables were compared using the χ^2^ tests (or Fisher’s exact where necessary), and continuous variables using the independent samples *t*-test or the Mann–Whitney *U* test, as appropriate. Pearson’s correlation coefficient was used to examine associations between continuous parameters. Univariable logistic regression analysis was performed to evaluate the association between clinical parameters and the presence of pressure reversal. Kaplan–Meier survival curves were estimated for the LV-IVPG variables using medians, and the log rank test was used to assess differences. Unadjusted and adjusted Cox proportional hazards regression analyses were performed to evaluate the hazard ratio (HR) and 95% confidence interval (included as continuous variables). Covariates known to be of prognostic value in DCM patients [age, sex, New York Heart Association (NYHA) class, LVEDVi, LVEF, presence of LGE, LA strain, and LV-GLS] were univariably tested, and when *P* < 0.1, included in the adjusted models. Statistical analysis was performed using SPSS 26.0 (IBM Corp., Armon, NY, USA) software. A two-tailed *P* < 0.05 was considered statistically significant.

## Results

A total of 447 patients (mean age 55 ± 13 years and 60% male) were included. Clinical characteristics are summarized in *Table 1*. Eighteen percent of patients had a history of HF hospitalization and 14% presented with NYHA class ≥3. The median LVEF was 37 [26–45]%, LA volume index (LAVI) was 26 [19–38]mL/m^2^, and LV-GLS was −14 [−10 to −18]%. Non-ischaemic LGE was observed in 172 (39%) patients.

**Table 1:** Clinical and CMR characteristics of patient population

	All patients (*n* = 447)	Patients without pressure reversal in ‘B’ (*n* = 279)	Patients with pressure reversal in ‘B’ (*n* = 168)	*P*-value^a^
Demographics				
Age (years)	55 ± 13	51 ± 13	59 ± 11	<0.001
Male sex (*n*)	266 (60%)	175 (63%)	91 (54%)	0.074
Body mass index (kg/m^2^)	26 [23–29]	26 [24–30]	25 [23–29]	0.115
Hypertension (*n*)	130 (29%)	75 (27%)	55 (33%)	0.187
Hypercholesterolaemia (*n*)	72 (16%)	44 (16%)	28 (17%)	0.803
Diabetes mellitus (*n*)	50 (11%)	31 (11%)	19 (11%)	0.949
Atrial fibrillation	40 (9%)	25 (9%)	15 (9%)	0.991
Heart failure hospitalization (*n*)	79 (18%)	50 (18%)	29 (17%)	0.860
NYHA class ≥3 (*n*)	64 (14%)	36 (13%)	28 (17%)	0.271
QRS >120 ms (*n*)	129 (29%)	64 (23%)	65 (39%)	<0.001
Typical LBBB (*n*)	155 (35%)	106 (33%)	49 (58%)	<0.001
NT-proBNP (pg/mL)^b^	79 [24–240]	51 [13–176]	139 [48–327]	<0.001
Use of diuretics (*n*)	189 (42%)	97 (35%)	92 (55%)	<0.001
CMR baselines				
LVEDV-indexed (mL/m^2^)	121 [101–150]	116 [94–138]	140 [111–163]	<0.001
LVEF (%)	37 [26–45]	41 [30–48]	29 [21–37]	<0.001
RVEDV-indexed (mL/m^2^)^c^	82 [69–99]	84 [71–101]	78 [65–95]	0.005
RVEF (%)^c^	51 [44–57]	51 [45–57]	51 [41–57]	0.418
LAVI (mL/m^2^)	26 [19–38]	24 [18–38]	28 [21–40]	0.090
LGE presence	172 (39%)	100 (36%)	72 (43%)	0.140
LV-GLS (%)	−14 ± 5	−16 [−11–−19]	−12 [−9–−15]	<0.001
LA reservoir strain (%)	26 ± 11	28 [20–34]	24 [17–30]	0.003
LA conduit strain (%)	12 ± 8	13 [8–20]	9 [5–14]	<0.001
LA booster strain (%)	14 ± 6	13 [8–17]	14 [10–18]	0.132
LV-IVPG analysis				
Overall IVPG	10 [7–13]	11 [8–14]	8 [6–11]	<0.001
Systolic ejection ‘A’	14.2 [9.7–18.9]	16.1 [10.6–20.5]	11.8 [8.4–16.0]	<0.001
Systolic–diastolic transition ‘B’	−4.3 [−3.1–−6.1]	−4.8 [−3.5–−6.5]	−3.9 [−3.0–−5.4]	0.001
Systolic slowdown ‘B1’	−4.2 [−2.8–−5.8]	−4.3 [−3.0–−5.8]	−3.9 [−2.7–−5.8]	0.280
Diastolic suction ‘B2’	−4.5 [−2.9–−6.9]	−4.8 [−3.2–−7.9]	−3.7 [−2.7–−5.5]	<0.001
E-wave deceleration ‘C’^d^	3.8 [2.3–6.1]	4.5 [3.0–7.0]	2.6 [1.3–4.2]	<0.001
A-wave acceleration ‘D’	−1.8 [−0.9–−3.0]	−1.8 [−1.0–−3.2]	−1.7 [−0.9–−2.8]	0.543

EDV, end-diastolic volume; EF, ejection fraction; ESV, end-systolic volume; GLS, global longitudinal strain; IVPG, intraventricular pressure gradient; LGE, late gadolinium enhancement; LV, left ventricular; LA, left atrium; LAVI, LA volume-indexed; LBBB, left bundle branch block; NYHA, New York Heart Association; RV, right ventricular.

Difference between patients with and without pressure reversal in ‘B’.

Available in 326 patients (73%).

Missing in 4 patients.

Missing in 2 patients.

### LV-IVPG patterns in the DCM population

**Figure 2:**
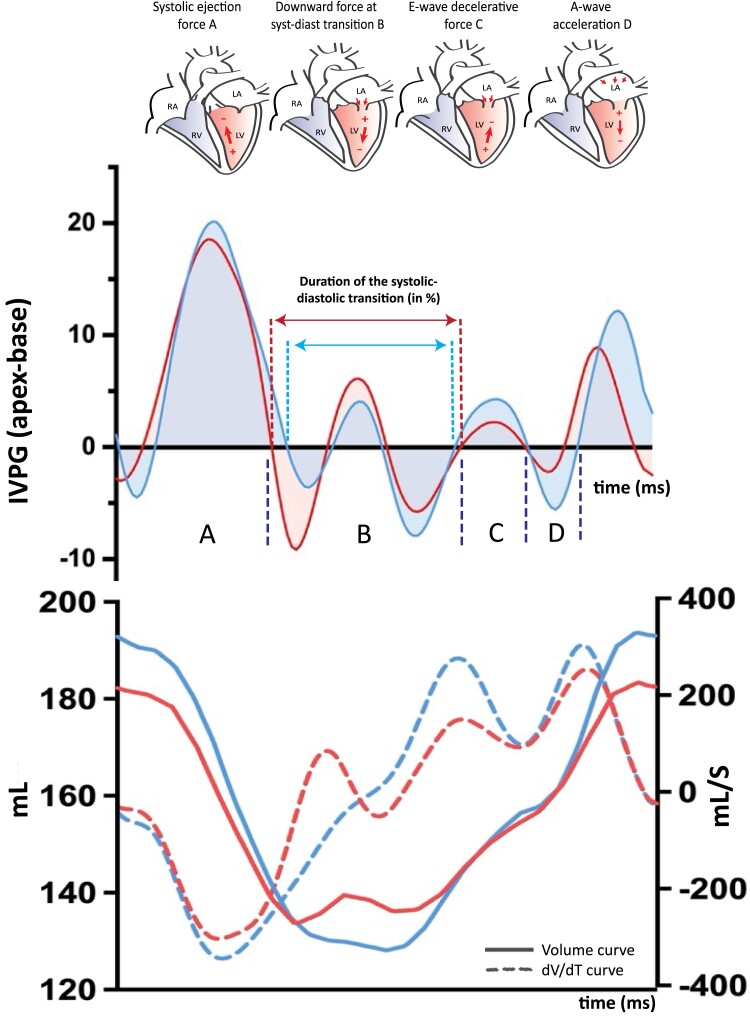
LV-IVPG analysis in two patients with pressure reversal in the B-wave. In 168 patients (38% of the total study population), there was a pressure reversal visible during the systolic–diastolic transition ‘B’ (blue lines). This means that the IVPG is reversed from base–apex (negative wave), driving early LV filling, to an IVPG directed from apex–base (positive wave), hampering cardiac blood inflow. These patients had higher NT-proBNP levels and worse overall LV-functional parameters compared to patients without pressure reversal. In 19 patients (14%), pressure reversal was so severe that it resulted in blood flow reversal, directed towards the base (red lines), as depicted in the *dV*/*dT* and volume curve at the bottom of the graph (red lines).

The LV-IVPG analysis was feasible in all 447 patients (*Table 1*). A temporary reversal of IVPG from base–apex to apex–base during the systolic–diastolic transition ‘B’ (*Figure 2*) occurred in 168 patients (38%), indicating that apical pressures are temporarily higher than basal pressures, hampering diastolic filling. Patients with this so-called pressure reversal during the B-wave had a significantly higher age, NT-proBNP levels, and use of diuretics compared to patients without reversal (*Table 1*). Furthermore, patients with pressure reversal had higher LVEDVi and worse LVEF, LV-GLS, and LA strain, compared to those without. In general, LV-IVPG parameters were also impaired in these patients, except for the systolic slowdown ‘B1’ and A-wave ‘D’ (atrial contraction). In most patients, this pressure reversal led to a prolonged diastolic–systolic transition and/or slower filling, but not to an actual reversal of the blood flow. In 24 of the 168 patients with pressure reversal (14%), it also led to actual reversal of the blood flow direction. Here, the blood flow directed from apex to base instead of base to apex (*Figure 2*, red curve). Higher age, ventricular conduction delay (QRS >120 ms), presence of LBBB, higher LVEDVi, and RVEDVi, lower LVEF, worse LV-GLS, LA reservoir, and conduit strain were univariably associated with presence of pressure reversal (see Supplementary data online, *Table S1*). Although patients with a LBBB had a pressure reversal in the systolic–diastolic transition ‘B’ more often (58% vs. 33%, *P* < 0.001), the other LV-IVPG parameters were comparable with patients without a LBBB (see Supplementary data online, *Table S2*).

### LV-IVPG predictors of outcome

In total, 66 patients (15%) reached the combined endpoint (HF hospitalization (*n* = 24), LTAs (*n* = 13), and sudden or cardiac death (*n* = 28)) during a median follow-up of 6 [4–9] years. There were no patients lost to follow-up. Age, sex, NYHA class ≥3, LVEF, LGE presence, LV-GLS, LAVI, and LA conduit strain were all univariable predictors of outcome in the total study population (see Supplementary data online, *Table S3*). Supplementary data online, *Table S4* shows that the LV-IVPG parameters are still of prognostic significance to predict sudden/cardiac death (*n* = 37) alone, or sudden/cardiac death and LTAs (*n* = 47) combined. Overall LV-IVPG, systolic ejection ‘A’, and E-wave deceleration ‘C’ were univariably associated with prognosis. E-wave deceleration ‘C’ remained predictive [HR 0.88 95% CI (0.80–0.98), *P* = 0.014, *Table 2*] in multivariable analysis, adjusted for all univariable predictive covariates.

**Table 2:** The adjusted predictive value of LV-IVPG analysis (adjusted for all univariable predictors with *P* < 0.100)

	All patients (*n* = 447)		Patients without pressure reversal in ‘B’^a^ (*n* = 279)		Patients with pressure reversal in ‘B’^b^ (*n* = 168)	
	HR [95% CI]	*P*-value	HR [95% CI]	HR [95% CI]	*P*-value	HR [95% CI]
Overall IVPG	0.96 [0.88–1.05]	0.351	0.91 [0.83–0.99]	0.033	—	—
Systolic ejection ‘A’	0.97 [0.91–1.03]	0.355	0.91 [0.86–0.96]	<0.001	—	—
Systolic–diastolic transition ‘B’	—	—	1.14 [0.97–1.33]	0.115	—	—
Reversal duration (% of cycle)	—	—	—	—	1.10 [0.99–1.22]	0.088
Flow reversal	—	—	—	—	2.57 [1.01–6.51]	0.047
E-wave deceleration ‘C’	0.88 [0.80–0.98]	0.014	0.83 [0.73–0.94]	0.003	—	—
A-wave acceleration ‘D’	—	—	—	—	—	—

Abbreviations as in *Table 1*.

Adjusted for age, NYHA class ≥3, LVEF, LGE, LV-GLS, LAVI, and LA conduit strain.

Adjusted for age, NYHA class ≥3, and LA conduit strain.

#### LV-IVPG predictors of outcome in patients with pressure reversal in systolic diastolic transition

In total, 22 of the 168 patients with pressure reversal in systolic–diastolic transition ‘B’ (13%) reached the primary combined endpoint. Although patients with pressure reversal had worse functional parameters (LVEF, LV-GLS, and LA strain) compared to those without, pressure reversal was not associated with a worse prognosis [HR 0.79 (0.47–1.32), *P* = 0.363]. Furthermore, none of the clinical or functional parameters predicted outcome in the subgroup of patients with pressure reversal, except for age (see Supplementary data online, *Table S3*). Next, we analysed if the severity of the pressure reversal was associated with outcome by quantifying the duration of the systolic–diastolic transition (as % of the total cycle) and the presence of reversal of blood flow direction (example in *Figure 2*, red curve). The duration of the systolic–diastolic transition univariably predicted worse outcome [HR 1.13 (1.03–1.24), *P* = 0.013]. Patients with a longer than median duration of the systolic–diastolic transition (>32%) had worse prognosis compared to patients with shorter durations (log rank test *P* = 0.003, Supplementary data online, *Figure S2*). The presence of reversal of blood flow direction from base–apex to apex–base predicted worse prognosis [HR 2.57 (1.01–6.51), *P* = 0.047] and remained associated after adjustment for age, NYHA class ≥3, and LA conduit strain (*Table 2*).

#### LV-IVPG predictors of outcome in patients without pressure reversal in systolic diastolic transition

**Figure 3:**
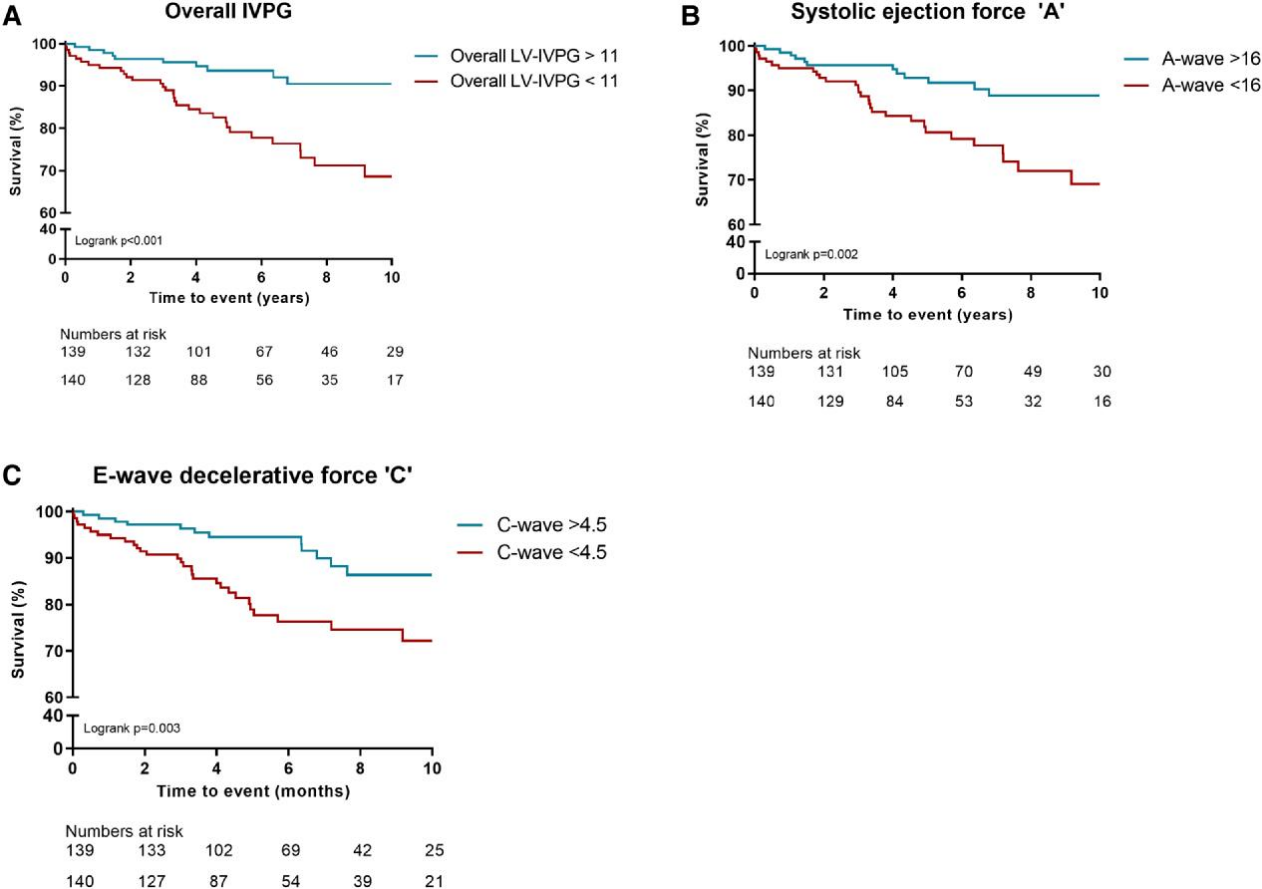
Kaplan–Meier event-free survival analysis of patients without pressure reversal during systolic–diastolic transition. In the absence of this pressure reversal pattern (*n* = 279), overall IVPG below the median of 11 had a worse outcome compared to patients with an overall IVPG >11 (log rank test *P* < 0.001) (A). Patients with a systolic ejection below the median of 16 had worse prognosis than patients with a systolic ejection >16 (log rank test *P* = 0.002) (B). An E-wave deceleration below the median of 4.5 had a significantly worse outcome compared to patients >4.5 (log rank test *P* = 0.003) (C).

In patients without pressure reversal in the systolic–diastolic transition, the primary outcome occurred in 44 patients (16%). NYHA class ≥3, LVEF, LGE presence, LV-GLS, LAVI, and LA conduit strain were univariably predictive of outcome in patients without pressure reversal. Additionally, all LV-IVPG parameters predicted outcome in this patient group (see Supplementary data online, *Table S3*), except for the atrial contraction ‘D’. After adjustment for all predictive covariates, overall LV-IVPG [HR 0.91 (0.83–0.99), *P* = 0.033], systolic ejection [HR 0.91 (0.86–0.96), *P* < 0.001], and the E-wave deceleration [HR 0.83 (0.73–0.94), *P* = 0.003] were independent predictors of outcome (*Table 2*). *Figure 3* displays the Kaplan–Meier event-free survival curves of overall LV-IVPG, systolic ejection, and E-wave deceleration, showing that patients with values below the median have significantly worse outcome than patients with better LV-IVPG values (log rank test *P* < 0.05 for all).

## Discussion

Fluid dynamics in the heart are of utmost importance for efficient and optimal cardiac function. As early as mid-20th century, invasive pressure measurements in both animal and human studies have found that pressure gradients between apex and base, created by ventricular relaxation and contraction, regulate cardiac blood flow.^4–7,14^ This study evaluates alterations of CMR-derived LV-IVPGs and their prognostic value in DCM patients. The main novelties are: (i) two visually distinctive LV-IVPG patterns in DCM patients occur, distinguished by the presence or absence of a pressure gradient reversal in the systolic–diastolic transition, and (ii) LV-IVPG parameters are of independent value to predict outcome in DCM patients, especially in the absence of the pressure gradient reversal pattern (*Graphical abstract*).

Echocardiography would be the preferred technique, as it is widely available and inexpensive, and the first line imaging modality.^15^ Standard echocardiographic measurements using the Bernoulli equation and peak velocities can calculate local pressure gradient differences across a valvular stenosis or LV outflow tract obstruction.^16^ However, this cannot be used to measure generalized pressure differences over a (non-flow obstructed) LV. Colour Doppler M-mode^17^ and particle image velocimetry^18^ are experimental echocardiographic methods to measure LV-IVPG. Unfortunately, they are not clinically applicable due to time expensive post-processing, complex acquisition, or the need of a contrastagent. 4D-flow CMR is the current gold standard of measuring LV-IVPG non-invasively.^19,20^ Though, it requires additional CMR acquisitions and time-expensive post-processing. The haemodynamic force analysis in this study uses standard cine images, only takes a few minutes of post-processing time, and is validated against 4D-flow CMR.^21^

### Different patterns of LV-IVPG in DCM patients

Temporary pressure gradient reversal during the transition from systole to diastole occurred in one-third of DCM patients. This IVPG shift from base–apex to apex–base indicates higher apical pressures than basal pressures, negatively affecting diastolic filling. Due to the novelty of this method, previous studies are scarce, and this is the first time that this pattern is introduced in HF patients. In our study, higher LVEDVi and RVEDVi, and ventricular conduction delay (QRS > 120 ms) were associated with this aberrant LV-IVPG pattern. It has been previously described that diastolic dysfunction impairs LV-IVPG.^9,22^ The association of higher RVEDVi with the IVPG reversal pattern might be explained by ventricular interdependence as is seen in a previous pulmonary hypertension study of ours.^13^ Ventricular conduction delay (QRS > 120 ms), indicating dyssynchrony, was associated with an IVPG shift as well. Dyssynchrony is known to influence LV-IVPGs, as is described in a HF study in which the presence of a LBBB altered LV-IVPGs to a more orthogonal direction in early diastole.^23^

In small pilot studies, DCM patients had aberrant flow patterns, showing larger vortex areas, and early diastolic flow directed more orthogonally to the main flow direction in comparison to healthy controls.^20,24^ It is speculated that aberrant flow patterns not only hamper optimal filling but also cause increased wall stress, inducing further adverse cardiac remodelling.^20,25^ This confirms our observation of pressure reversal patterns in DCM patients negatively affecting diastolic filling.

### LV-IVPGs are associated with prognosis

Interestingly, the observed presence of pressure reversal in the systolic–diastolic transition was not associated with worse outcome, although these patients had higher NT-proBNP levels and worse cardiac functional indices such as LVEF, LV-GLS, and LA strain. This might be explained by the heterogeneity of this group. Other factors might play a role as well, such as ventricular conduction delay (QRS duration) and higher RVEDVi, which were associated with a temporary pressure reversal, but not with worse outcome. Not the presence, but more the severity is associated with worse outcome. A longer duration of the systolic–diastolic transition was univariably associated with adverse prognosis. Reversal of blood flow direction was associated with adverse prognosis, independent of clinical covariates such as age, NYHA class ≥3, and LA conduit strain. This indicates that this pattern is not only related to more severe heart failure or age, but is of independent prognostic value.

In patients without pressure reversal, impaired overall LV-IVPG, systolic ejection, and E-wave deceleration were strong predictors of adverse outcome, independent of all evaluated clinical and imaging parameters (age, sex, NYHA class ≥ 3, LVEF, LGE presence, LV-GLS, LAVI, and LA conduit strain).

### Clinical implications of LV-IVPG analysis

LV-IVPG analysis provides insight in fluid dynamics without the need of an invasive procedure, and therefore help our understanding of the pathophysiological processes in various cardiac diseases. The ability of LV-IVPG to evaluate diastolic filling is of utmost importance, since diastolic LV functional parameters on CMR are lacking. Post-processing analysis takes a few minutes, only requiring manual drawing of valve diameters and LV endocardial contours in end-systole and end-diastole of the three long-axis cine images. In addition, inter- and intra-observer variability is very limited.^13^ In our study, LV-IVPG analysis revealed different patterns in DCM patients, and several parameters were predictive of adverse outcome, independent of known clinical and imaging predictors such as LGE, LA strain, and LV-GLS which have been extensively investigated as important prognostic markers in DCM as well.^26–28^ Therefore, this analysis could potentially improve risk stratification and clinical management.

### Limitations

This is the first large prospective study evaluating LV-IVPG curves, patterns, and their prognostic value in DCM patients. Although promising, these results need to be confirmed in other DCM studies to see whether these results are reproducible and consistent. Only patients with CMR, with all three long-axis cine images in sinus rhythm available, could be included in this study. Patients with atrial fibrillation during the CMR were excluded in this study, and not all patients included in the Maastricht Cardiomyopathy registry underwent CMR imaging in the diagnostic work-up due to various reasons (logistic and/or patient related, such as claustrophobia, etc.), which might have introduced a selection bias.

## Conclusions

LV-IVPG analysis revealed two visually distinctive LV-IVPG patterns in DCM patients. A temporary pressure gradient reversal in systolic–diastolic transition opposite to the LA, hampering diastolic filling, occurred in one-third of patients. In a subset of these patients, this pressure gradient reversal was so severe that it caused a reversal of blood flow direction, which was independently associated with worse outcome. In patients without pressure reversal, lower overall LV-IVPG, impaired systolic ejection, and impaired E-wave deceleration (end of passive filling) predicted adverse outcome, independent of all evaluated clinical and imaging covariates.

## Supplementary data

Detailed CMR acquisition and analysis protocols are described in the Supplementary data online, *Methods*. Supplementary data online, *Table S1* shows the univariable predictors of pressure reversal during systolic–diastolic transition ‘B’. Supplementary data online, *Table S2* shows univariable predictors of outcome in total study population, patients without reversal of pressure in systolic–diastolic transition ‘B’ and patients with reversal of pressure in systolic–diastolic transition ‘B’. The study flow-chart is presented in Supplementary data online, *Figure S1*. Supplementary data online, *Figure S2* shows Kaplan–Meier event-free survival analysis of duration of the systolic–diastolic transition ‘B’ (in %) in patients with pressure reversal in systolic–diastolic transition.

Supplementary data is available at *European Heart Journal - Cardiovascular Imaging* online.

## Supplementary Material

jead083_Supplementary_Data

## Data Availability

The data underlying this article will be shared on reasonable request to the corresponding author.
