# Supplementary material for: CMR-derived left ventricular intraventricular pressure gradients identify different patterns associated with prognosis in dilated cardiomyopathy
Source: Eur Heart J Cardiovasc Imaging. 2023 May 3;24(9):1231–40. doi: 10.1093/ehjci/jead083 (PMC10445254; doi:10.1093/ehjci/jead083)
Supplement: jead083_Supplementary_Data [file jead083_supplementary_data.zip › Supplementary data-clean version.docx]

**Supplemental methods**

*CMR acquisition and analysis*

CMR imaging was performed on a 1.5T system (Intera, Philips Medical Systems, Best, The Netherlands), including cine and late gadolinium enhancement (LGE) imaging in identical slice positions in short axis (covering the entire LV) and long axis orientations (including 2- 3- and 4-chamber). Balanced steady-state free precession cine images were acquired during end-expiratory breath holds, with the following typical sequence parameters: repetition time 3.0-3.5ms, echo time 1.5-1.8ms, flip angle 60°, temporal resolution <50ms. All post-processing analyses were performed using Medis Suite MR software (Medis Medical Imaging bv, Leiden, The Netherlands). LV and right ventricular (RV) volumes, and LV mass were measured, and LV/RV EF were calculated. The LGE images were obtained 10-15 minutes after administration of an intravenous bolus of a gadolinium-based contrast agent (Gadovist, Bayer Healthcare, Berlin, Germany), using a 2D segmented inversion-recovery gradient-echo pulse sequence. First, the presence of LGE was visually assessed, and if present, LGE was quantified using the full-width at half maximum technique (in grams and expressed as percentage of the total LV mass) ^1^. Non-specific RV insertion point LGE was excluded from the LGE analysis.

LV global longitudinal strain (GLS) was measured on the long-axis 2-, 3- and 4-chamber cine images and LA strain on the long-axis 2- and 4-chamber cine images, using Medis QStrain software (Medis Medical Imaging bv, version 2.0.48.8, Leiden, the Netherlands). Endocardial contours were manually drawn at the end-systolic and the end-diastolic phase, after which the software automatically tracks the contours in the remaining frames and calculates myocardial longitudinal shortening: the average LV-GLS (%), and LA reservoir (passive LA filling during LV systole), conduit (passive filling of blood from the LA to the LV), and booster strain (atrial contraction).

*CMR LV-IVPG analysis*

Pressure gradients are pressure differences between apex and base. These differences regulate inflow (early and late diastole) and outflow (LV contraction) of intracardiac blood. Pressure gradients are generated by myocardial properties, such as ventricular contraction (ejection phase), ventricular relaxation and recoil (early filling), atrial contraction (late filling), and the pressure in the large vessels. Hemodynamic force analysis is a method to calculate the global vector, averaged over the entire LV, of these intraventricular pressure gradients between apex and base throughout the cardiac cycle. To this aim, a 3D-LV model is automatically reconstructed from the feature tracking data of the long axis cine images, combined with the inflow through the manually drawn mitral valve area (in diastole) and outflow towards the manually drawn aortic valve area (in systole). By using the myocardial movement and velocity of the 3D-LV model in combination with the blood velocity over the valves, the integral of pressure gradient over the LV volume is calculated (the hemodynamic force). This integrated pressure gradient is then normalized for LV volume and divided by the specific gravity of blood, which makes it a dimensionless measure, to be interpreted as % of force of gravity.

Mathematical formula of the LV-IVPG analysis:

By definition, the global hemodynamic force vector is obtained by summing up the value of the pressure gradient at all points inside the ventricular volume $V(t)$

$$\boldsymbol{F}\left( t \right)=\iiint_{V(t)}^{-} \nabla p dV.$$

This volumetric integral can be rewritten in a different form with the aid of the Navier-Stokes equation, which uses the blood velocity, $\boldsymbol{v}$, at all points in time inside the LV volume, taking the blood density ($\rho=1.0kg/L$) into account

$$\boldsymbol{F}\left( t \right)=\rho\iiint_{V(t)}^{-} \left( \frac{\partial\boldsymbol{v}}{\partial t}+\boldsymbol{v}\cdot\nabla\boldsymbol{v} \right)dV.$$

This expression can be further transformed, with the aid of the Gauss theorem, in a form that uses the values of velocity at all points on the surface $S\left( t \right)$ that surrounds the LV volume

$$\boldsymbol{F}\left( t \right)=\rho\int\int_{S\left( t \right)}^{-} \left[ \boldsymbol{x}\left( \frac{\partial\boldsymbol{v}}{\partial t}\cdot\boldsymbol{n} \right)+\boldsymbol{v} (\boldsymbol{v}\cdot\boldsymbol{n}) \right]dS$$

where $\boldsymbol{x}$ are the three-dimensional coordinate of the points on the LV surface and $\boldsymbol{n}$ is the normal unit vector. This is the formula used in the present study.

In short, the global hemodynamic force vector represents the global force exchanged between blood volume and the endocardium, at a certain time point. This calculation involves the force vector integral over the LV boundary instead of the internal volume. Thus it only needs the velocity over the endocardial boundary (derived from the myocardial movement using the same feature tracking results used for strain and strain rate analysis) and the blood velocity across the valves (which is calculated from the volumetric changes of the LV and the valve area, using the conservation of mass principle). The three-dimensional surface is reconstructed by combination of the three apical views. The physical rationale is described in more detail in recent articles of Vallelonga et al.^2^ and Pedrizzetti et al. ^3^

The IVPG-time curve represents the vector of IVPG directed from apex to base (reflected by a *positive wave*), or from base to apex (reflected by a *negative wave*) throughout the cardiac cycle (**Figure 2**). The first wave (**A**) represents LV contraction, a positive LV-IVPG from apex to base (depicted as a *positive wave*), which is called the systolic ejection force. The second wave (**B**) represents the systolic slowdown and transition from systole to diastole, a pressure gradient from base-apex (depicted as a *negative wave*), consisting of phase B1 and B2. B1 starts at the end of systole, representing the systolic slowdown. When the LV relaxes and unwinds, the LV pressure falls below the pressure in the outflow tract, closing the aortic valve. This marks the beginning of the B2 phase, which begins with the isovolumic relaxation, until the LV pressure falls below the LA pressure, opening the mitral valve, and leading to an acceleration of blood inflow from the LA to the LV (base-apex, depicted as a *negative wave*): the diastolic suction. The third wave (**C**) coincides with the E-wave decelerative force and represents the slowing blood flow rate at the end of the passive filling phase, resulting from increasing LV pressures (due to LV filling) until the LV pressure is higher than LA pressure (apex-base IVPG, depicted as a *positive wave*). Next, the pressure between LA and LV equilibrates (diastasis), marking the end of the early filling phase. The fourth wave (**D**) coincides with the A-wave acceleration force that represents the late filling phase, when atrial contraction leads to increased LA pressures above LV pressures (base-apex, depicted as a *negative wave*). The overall LV-IVPG between apex-base (throughout the whole cardiac cycle), A-, B-, C- and D-waves evaluated in this study are illustrated in **Figure 2** (modified from Vos et al. ^4^). All CMR analyses were performed by two trained independent investigators (JV and AR), blinded to outcome and supervised by a level III CMR physician with >15 years of experience (RN).

**References**

1. Flett AS, Hasleton J, Cook C, Hausenloy D, Quarta G, Ariti C, et al. Evaluation of techniques for the quantification of myocardial scar of differing etiology using cardiac magnetic resonance. *JACC Cardiovasc Imaging*. 2011;**4**(2):150-6.

2. Vallelonga F, Airale L, Tonti G, Argulian E, Milan A, Narula J, et al. Introduction to Hemodynamic Forces Analysis: Moving Into the New Frontier of Cardiac Deformation Analysis. *Journal of the American Heart Association*. 2021;**10**(24):e023417.

3. Pedrizzetti G. On the computation of hemodynamic forces in the heart chambers. *Journal of biomechanics*. 2019;**95**:109323.

4. Vos JL, Leiner T, van Dijk APJ, Pedrizzetti G, Alenezi F, Rodwell L, et al. Cardiovascular magnetic resonance-derived left ventricular intraventricular pressure gradients among patients with precapillary pulmonary hypertension. *European heart journal cardiovascular Imaging*. 2022.

| **Table S1.** Interobserver variability of LV-IVPG parameters | | |
| --- | --- | --- |
| Variables | **Interobserver variability** | |
|  | ICC (95% CI) | p-value |
| **LV intraventricular pressure gradient analysis (apex-base)** |  |  |
| IVPG during total cardiac cycle | 0.95 (0.89-0.98) | <0.001 |
| Systolic ejection force ‘A’ | 0.86 (0.70-0.94) | <0.001 |
| Downward force at systolic-diastolic transition ‘B’ | 0.78 (0.70-0.95) | <0.001 |
| E-wave decelerative force ‘C’ | 0.87 (0.70-0.94) | <0.001 |
| A-wave acceleration force ‘D’ | 0.95 (0.88-0.98) | <0.001 |
| *Abbreviations:* IVPG, intraventricular pressure gradient; LV, left ventricular | | |

| **Table S2.** Univariable predictors of pressure reversal during systolic-diastolic transition ‘B’ | | |
| --- | --- | --- |
| Variables | OR [95% CI] | p-value* |
| Age (years) | 1.06 [1.04-11.08] | <0.001 |
| Male sex (n) | 0.70 [0.48-1.04] | 0.075 |
| NYHA class ≥3 | 1.35 [0.79-2.31] | 0.272 |
| QRS duration (ms) | 1.02 [1.01-1.03] | <0.001 |
| QRS >120 ms (n) | 2.09 [1.37-3.19] | <0.001 |
| Presence of LBBB (n) | 2.75 [1.69-4.48] | <0.001 |
| NT-proBNP (pg/mL) | 1.00 [1.00-1.00] | 0.421 |
| Echocardiographic E/A ratio | 0.59 [0.40-0.87] | 0.007 |
| LVEDV-indexed (mL/m^2^) | 1.01 [1.00-1.02] | <0.001 |
| LVEF (%) | 0.93 [0.92-0.95] | <0.001 |
| LV-GLS | 1.17 [1.12-1.22] | <0.001 |
| LA reservoir strain | 0.98 [0.96-0.99] | 0.010 |
| LA conduit strain | 1.03 [0.99-1.06] | 0.077 |
| RVEDV-indexed (mL/m^2^) | 0.99 [0.98-1.00] | 0.011 |
| RVEF (%) | 0.99 [0.98-1.00] | 0.307 |
| *Abbreviations:* EDV, end-diastolic volume; EF, ejection fraction; ESV, end-systolic volume; GLS, global longitudinal strain; LBBB, left bundle branch block; LV, left ventricular; LA, left atrium; NYHA, New York Heart Association; RV, right ventricular. | | |

| **Table S3.** LV-IVPG characteristics of patient population with and without a LBBB | | | | |
| --- | --- | --- | --- | --- |
|  | Patients with a LBBB (n=85) | Patients without a typical LBBB (n=320)^a^ |  | p-value |
| **LV-IVPG analysis (apex-base)** |  |  | |  |
| IVPG during total cardiac cycle | 10 [7-14] | 10 [7-13] |  | 0.553 |
| Systolic ejection force ‘A’ | 14 [10-19] | 14 [10-19] |  | 0.965 |
| Downward force at systolic-diastolic transition ‘B’ | -4.2 [-3.0 - -6.7] | -4.3 [-3.1 - -5.8] |  | 0.811 |
| Pressure reversal in the systolic-diastolic transition ‘B’ | 49 (58%) | 106 (33%) |  | <0.001 |
| Systolic slowdown force ‘B1’ | -4.1 [-2.6 - -6.1] | -4.1 [-2.8 - -5.6] |  | 0.562 |
| Diastolic suction force ‘B2’ | -4.5 [-3.1 - -7.0] | -4.5 [-2.9 - -6.8] |  | 0.601 |
| E-wave decelerative force ‘C’ ^d^ | 3.3 [1.0-5.9] | 4.0 [2.5-6.0] |  | 0.026 |
| A-wave acceleration force ‘D’ | -1.7 [-0.5 - -2.8] | -1.8 [-1.0 - -2.9] |  | 0.587 |
| *Abbreviations:* IVPG, intraventricular pressure gradient; LBBB, left bundle branch block; LV, left ventricular  ^a^ Patients with an intraventricular delay (QRS duration >120ms) without a typical LBBB (n=42) have been excluded from this analysis. | | | | |

| **Table S4.** Univariable predictors of outcome in total study population, patients without and patients with reversal of pressure in systolic-diastolic transition ‘B’ | | | | | | |
| --- | --- | --- | --- | --- | --- | --- |
| Variables | All patients (n=447) | | Patients without pressure reversal in ‘B’ (n=168) | | Patients with pressure reversal in ‘B’  (n=279) | |
|  | HR [95% CI] | p-value | HR [95% CI] | p-value | HR [95% CI] | p-value |
| Age | 1.03 [1.01-1.05] | 0.012 | 1.02 [1.00-1.05] | 0.060 | 1.05 [1.01-1.10] | 0.019 |
| Male sex | 1.10 [0.65-1.86] | 0.729 | 0.97 [0.51-1.86] | 0.932 | 1.34 [0.55-3.28] | 0.522 |
| NYHA class ≥3 | 2.46 [1.43-4.23] | 0.001 | 2.53 [1.27-5.02] | 0.008 | 2.37 [0.97-5.82] | 0.059 |
| LVEF | 0.97 [0.95-0.99] | 0.004 | 0.96 [0.94-0.98] | <0.001 | 0.99 [0.95-1.03] | 0.716 |
| LGE presence | 2.74 [1.66-4.52] | <0.001 | 3.24 [1.74-6.04] | <0.001 | 2.00 [0.85-4.68] | 0.111 |
| LV global longitudinal strain | 1.08 [1.02-1.13] | 0.004 | 1.12 [1.05-1.18] | <0.001 | 1.03 [0.93-1.13] | 0.634 |
| LAVI | 1.02 [1.01-1.04] | <0.001 | 1.03 [1.02-1.04] | <0.001 | 1.00 [0.97-1.03] | 0.990 |
| LA conduit strain | 0.92 [0.890-0.96] | <0.001 | 0.92 [0.88-0.96] | <0.001 | 0.92 [0.84-1.00] | 0.050 |
|  |  |  |  |  |  |  |
| **LV intraventricular pressure gradient analysis (apex-base)** | | |  |  |  |  |
| IVPG during total cardiac cycle | 0.90 [0.85-0.96] | 0.002 | 0.85 [0.79-0.93] | <0.001 | 0.98 [0.89-1.09] | 0.737 |
| Systolic ejection force ‘A’ | 0.93 [0.89-0.97] | 0.001 | 0.90 [0.85-0.95] | <0.001 | 0.98 [0.91-1.05] | 0.546 |
| Downward force at systolic-diastolic transition ‘B’ | 1.09 [0.98-1.22] | 0.112 | 1.20 [1.04-1.38] | 0.013 | 0.91 [0.77-1.09] | 0.299 |
| Pressure reversal in ‘B’ | 0.79 (0.47-1.32) | 0.363 | - | - | - | - |
| B-wave reversal duration (% of cycle duration) | - | - | - | - | 1.13 [1.03-1.24] | 0.013 |
| Flow reversal | - | - | - | - | 2.91 [1.16-7.32] | 0.023 |
| E-wave decelerative force ‘C’ | 0.88 [0.80-0.97] | 0.009 | 0.82 [0.73-0.93] | 0.002 | 0.93 [0.77-1.13] | 0.452 |
| A-wave acceleration force ‘D’ | 0.98 [0.86-1.13] | 0.811 | 1.02 [0.86-1.21] | 0.841 | 0.91 [0.73-1.14] | 0.411 |
| *Abbreviations:* EF, ejection fraction; IVPG, intraventricular pressure gradient; LGE, late gadolinium enhancement; LV, left ventricular; LA, left atrium; LAVI, LA volume-indexed; NYHA, New York Heart Association | | | | | | |

| **Table S5.** Univariable analysis of the LV-IVPG parameters with sudden/cardiac death, and a combined endpoint of sudden/cardiac death with life-threatening arrythmias in the total DCM study population | | | | |
| --- | --- | --- | --- | --- |
| Variables | Sudden/cardiac death (n=37) | | Sudden/ cardiac death or life-threatening arrythmias (n=47) | |
|  | HR [95% CI] | p-value | HR [95% CI] | p-value |
| **LV intraventricular pressure gradient analysis (apex-base)** |  |  |  |  |
| IVPG during total cardiac cycle | 0.90 [0.83-0.98] | 0.008 | 0.89 [0.83-0.96] | 0.002 |
| Systolic ejection force ‘A’ | 0.92 [0.87-0.97] | 0.004 | 0.91 [0.87-0.96] | <0.001 |
| Downward force at systolic-diastolic transition ‘B’ | 1.15 [0.99-.134] | 0.071 | 1.15 [1.15-1.32] | 0.042 |
| E-wave decelerative force ‘C’ | 0.85 [0.75-0.97] | 0.016 | 0.86 [0.77-0.96] | 0.010 |
| A-wave acceleration force ‘D’ | 0.94 [0.79-1.23] | 0.500 | 1.02 [0.85-1.22] | 0.853 |
| *Abbreviations:* IVPG, intraventricular pressure gradient; LV, left ventricular | | | | |

**
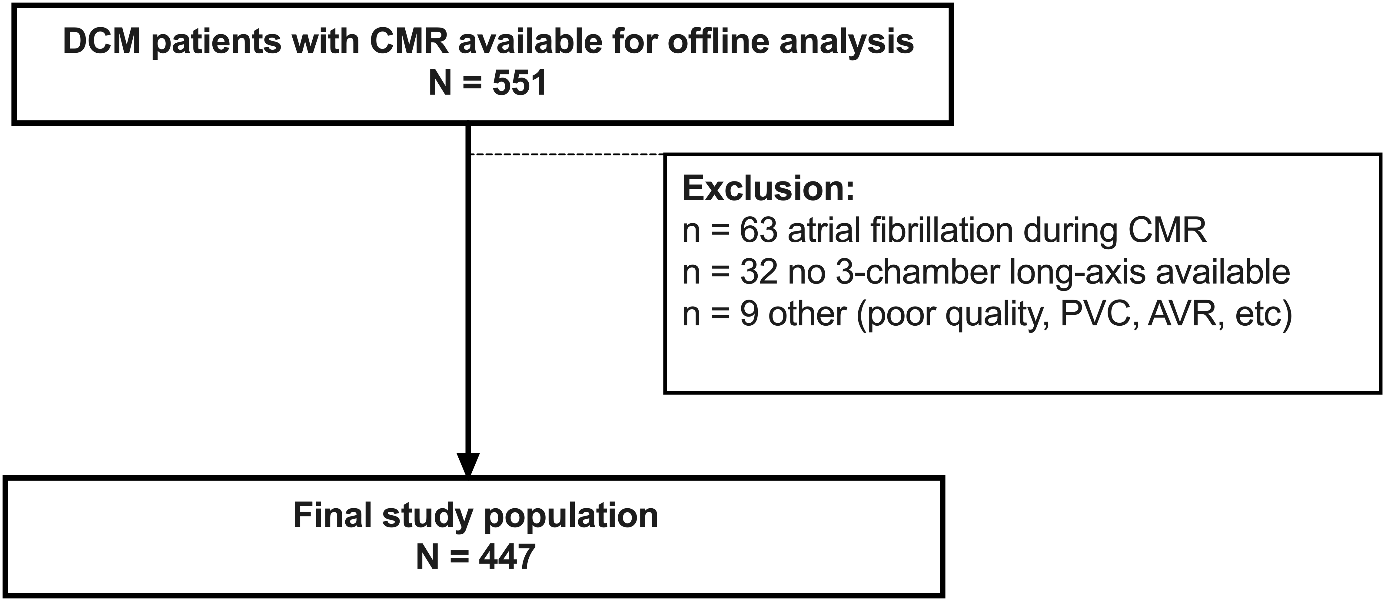
Figure S1:** Flow-chart

**Figure legend:** DCM patients included in the Maastricht Cardiomyopathy Registry were selected with available long-axis cines (3-, 4-, and 2-chamber views). Patients were excluded if they had atrial fibrillation during the CMR scan (n=63). Nine patients were excluded due to other reasons (poor quality, PVC, AVR etc.). A total of 447 patients was included.

*Abbreviations:* AVR, aortic valve replacement; CMR, cardiac magnetic resonance; DCM, dilated cardiomyopathy; PVC, premature ventricular complex


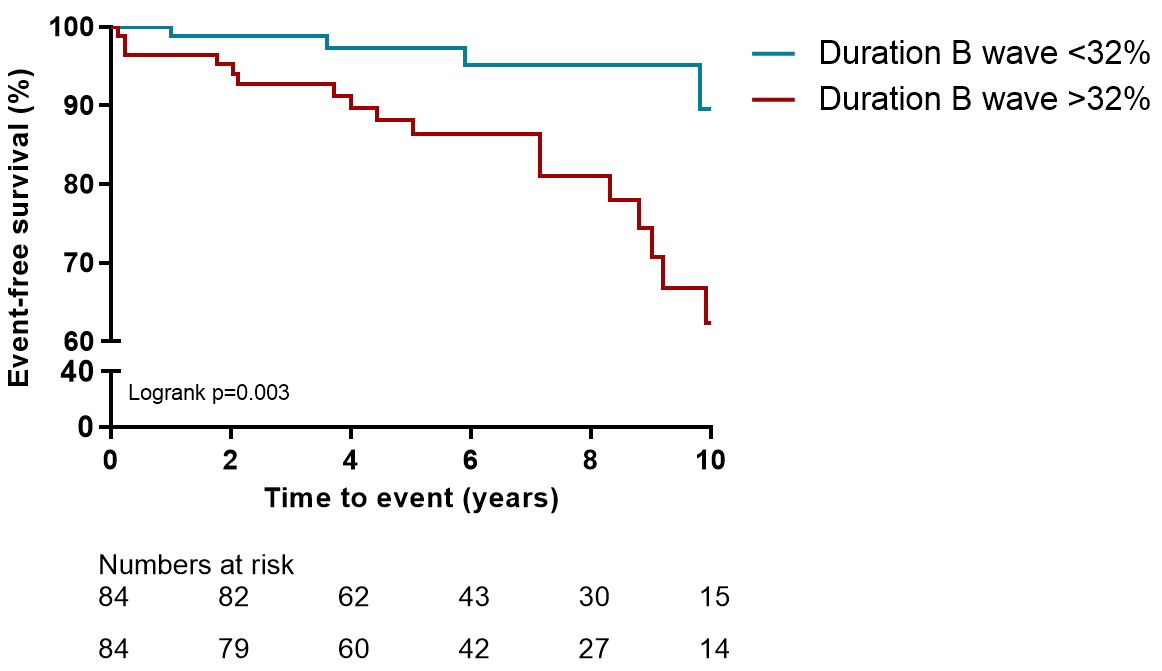
**Figure S2.** Kaplan-Meier event-free survival analysis of duration of the systolic-diastolic transition ‘B’ (in %)

**Figure legend:** Patients with pressure reversal in systolic-diastolic transition ‘B’ (n=168) with a systolic-diastolic transition duration above the median (>32% as % of the total cycle) had a worse prognosis compared to patients with shorter durations (<32%, Log rank test p=0.003).
